# Report of Johnson Co. Board of Censors

**Published:** 1887-03

**Authors:** F. C. Osborn

**Affiliations:** City Health Officer


### REPORT OF THE BOARD OF CENSORS OF THE JOHNSON COUNTY MEDICAL SOCIETY, IN THEIR CAPACITY OF COMMITTEE OF PUBLIC HEALTH.

For Daniel's Texas Medical Journal.

During the summer of 1884 the Board of Censors presented a report of their labors to the Medical Society, and embodied in it a memorial to the city council, pleading for ordinances for the establishment of books of registration for the births and deaths within the city limits, and were gratified to find their prayers successful.

The ordinances took effect in October, 1884 and the following statistics exhibit the vital and mortuary tables up to December 31, 1886, a period of 27 months therein included.

The population of the city, the census of which was also taken by the Board of Censors, amounted to above 5,000 inhabitants, and the births as recorded are as follows, namely:

| | |
|---|---|
| Total births | 174 |
| White | 165 |
| Colored | 9 |
| Males | 85 |
| Females | 89 |
| Ward No. 1 | 52 |
| Ward No. 2 | 70 |
| Ward No. 3 | 49 |
| Total | 171 |

The discrepancy is owing to failure to report the wards.

The register of deaths is as follows, viz:

| | |
|---|---|
| Total deaths | 125 |
| White | 120 |
| Colored | 5 |
| Males | 56 |
| Females | 60 |
| Sex not reported | 9 |
| Ward No. 1 | 40 |
| Ward No. 2 | 46 |
| Ward No. 3 | 39 |
| Total | 125 |

The following table includes the causes of death, viz:

| | |
|---|---|
| Paralysis of the Heart | 2 |
| Inanition | 8 |
| Malformed | 2 |
| Pneumonia | 8 |
| Œdema Glottidis | 1 |
| Congestion of the Brain | 3 |
| Still born | 7 |
| Peritonitis | 2 |
| Meningitis | 1 |
| Catarrhal Fever | 2 |
| Pulmonary Consumption | 5 |
| Typhoid Fever | 9 |
| Effects of Wound | 1 |
| Visceral Congestion | 1 |
| Gastritis | 1 |
| Paralysis | 3 |
| Abdominal Tumor | 1 |
| Phrenitis | 2 |
| Uremia | 1 |
| Congestion of the Lungs | 4 |
| Nephritis | 2 |
| Rheumatism | 2 |
| Dysentery | 4 |
| Debility | 3 |
| Opium Poisioning | 1 |
| Congestion | 2 |
| Puerperal Hemorrhage | 1 |
| Pelvic Abscess | 2 |
| Effusion on the Brain | 1 |
| Congestion of the Bowels | 2 |
| Heart Disease | 2 |
| Malarial Fever | 1 |
| Erysipelas | 1 |
| Tubercular Meningitis | 2 |
| Diarrhea | 2 |
| Cancrum Oris | 3 |
| Premature Birth | 1 |
| Capillary Bronchitis | 1 |
| Rotheln | 4 |
| Ovarian Dropsy | 1 |
| Acute Yellow Atrophy of the Liver | 2 |
| Cholera Infantum | 1 |
| Whooping Cough | 1 |
| Cyanosis | 2 |
| Ulceration of the Stomach | 1 |
| Hemorrhage of the Bowels | 1 |

| | |
|---|---|
| Septicemia | 1 |
| Congestion of the Stomach | 1 |
| Cause not reported | 13 |
| Total | 125 |
| Percentage per 1,000 | 1.25 |

Which is a very fair showing of the health of the city.

During the past winter season the Board of Censors, emboldened by their former success, again memorialized the city council for an ordinance for the registration of pestilential or infective diseases, and power of quarantine, and succeeded beyond their most sanguine expectations.

The city is now prepared to limit and stamp out any contagious diseases which may infest her borders.

The Board of Censors did not stop here, but are at this moment memorializing the County Commissioners to order the same system of registration under the supervision of a Board of Health for the county, the Board of Health to consist of the Board of Censors.

If the Board is successful in this effort, it is believed that Johnson county will lead all other counties in the State in this desirable sanitary reform.

F. C. OSBORN, M. D.
City Health Officer.

